# Kohonen Neural Network Investigation of the Effects of the Visual, Proprioceptive and Vestibular Systems to Balance in Young Healthy Adult Subjects

**DOI:** 10.3390/healthcare9091219

**Published:** 2021-09-16

**Authors:** Oseikhuemen Davis Ojie, Reza Saatchi

**Affiliations:** Industry and Innovation Research Institute (I2RI), College of Business, Technology and Engineering, Sheffield Hallam University, Sheffield S11WB, UK; ojieose@gmail.com

**Keywords:** mediolateral sway, anterior-posterior sway, balance-related sensory information, accelerometry, Kohonen neural network, balance dysfunction and diagnosis

## Abstract

Kohonen neural network (KNN) was used to investigate the effects of the visual, proprioceptive and vestibular systems using the sway information in the mediolateral (ML) and anterior-posterior (AP) directions, obtained from an inertial measurement unit, placed at the lower backs of 23 healthy adult subjects (10 males, 13 females, mean (standard deviation) age: 24.5 (4.0) years, height: 173.6 (6.8) centimeter, weight: 72.7 (9.9) kg). The measurements were based on the modified Clinical Test of Sensory Interaction and Balance (mCTSIB). KNN clustered the subjects’ time-domain sway measures by processing their sway’s root mean square position, velocity, and acceleration. Clustering effectiveness was established using external performance indicators such as purity, precision-recall, and F-measure. Differences in these measures, from the clustering of each mCTSIB condition with its condition, were used to extract information about the balance-related sensory systems, where smaller values indicated reduced sway differences. The results for the parameters of purity, precision, recall, and F-measure were higher in the AP direction as compared to the ML direction by 7.12%, 11.64%, 7.12%, and 9.50% respectively, with their differences statistically significant (*p* < 0.05) thus suggesting the related sensory systems affect majorly the AP direction sway as compared to the ML direction sway. Sway differences in the ML direction were lowest in the presence of the visual system. It was concluded that the effect of the visual system on the balance can be examined mostly by the ML sway while the proprioceptive and vestibular systems can be examined mostly by the AP direction sway.

## 1. Introduction

The maintenance of balance is mainly a combined process of the interaction of the central nervous system (CNS) and the balance-related sensory systems. In addition to other activities, in order to maintain balance, the CNS must correctly integrate information from the relevant sensory systems, i.e., the visual, proprioceptive, and vestibular systems [1]. These sensory systems act as sensors that contribute to a person’s balance and orientation by providing the necessary information pertaining to balance’s functionality. For example, the visual system is responsible for providing visual cues [2]; the proprioceptive system is responsible for providing information associated with the position and movement of the limbs and trunk [3]; the vestibular system is responsible for providing information associated with the spatial orientation and acceleration of the head [4]. Thus, the evaluation of the information from these systems is crucial in balance examinations.

The operations of the balance-related sensory systems could be assessed using the modified Clinical Test of Sensory Interaction and Balance (mCTSIB) [5,6,7,8]. The test consists of four conditions involving the subject being asked to (i) stand on a firm surface (i.e., ground) with eyes open, (ii) stand on a firm surface with eyes closed, (iii) stand on a flexible surface (e.g., a thick sponge) with eyes open, and (iv) stand on a flexible surface with eyes closed [8]. The first condition incorporates the three-balance-related sensory systems (i.e., visual, proprioceptive, and vestibular), the second condition incorporates the proprioceptive and vestibular systems, the third condition incorporates the visual and vestibular systems and the fourth condition incorporates only the vestibular system. Objectively, sway information can be obtained from the center of pressure (COP) and center of mass (COM) positions by examining the subject’s performance during mCTSIB and by using devices such as force platform and inertial measurement unit (IMU) [9,10,11]. Corresponding to the design characteristics, these devices are commonly used to produce sway information in two distinct body sway directions namely: the mediolateral (ML, i.e., side-to-side) direction and the anterior-posterior (AP, i.e., front-to-back) direction.

The sway directions (ML and/or AP) carry information that is crucial in understanding the behavioral patterns of the sensory systems which are necessary for diagnostic and monitoring purposes. These directions have been used in several studies to carry out postural sway analysis on various cohorts of subjects using varying analytical techniques. For example, utilizing the statistical technique of two way mixed design model and employing the root mean square (RMS) variables of COP displacement and velocity in the ML and AP directions, a comparison of postural strategies used to regain balance between older adults of ages 60 to 90 years, and younger adults of ages 20 to 40 years, undergoing a balance task i.e., standing quietly for 30 seconds, walking in place and then taking a lateral step and stand quietly (30 seconds) was carried out [12]. The work led to the suggestion that different strategies are used by older adults in the ML and AP directions and in order to recover balance after completing a lateral step, older adults prioritized postural stability in the AP direction [12]. Similarly, using mixed generalized linear models, a comparison between 22 adults with multiple sclerosis with mean age (standard deviation) of 56.3 (8.9) years and 22 correspondingly matched healthy adults with mean age (standard deviation) of 59.1 (7.1) years was conducted with the aim of assessing the differences between the groups in relation to their falls risk, strength, reactions, and directional control of balance [13]. The results obtained pertaining to the control of balance suggested that individuals with multiple sclerosis exhibited a greater overall COP motion in both the ML and AP directions [13]. Alternatively, the application of machine learning techniques such as clustering can be used for obtaining information pertaining to the examination of similarities and differences between groups by considering their clustering characteristics [14].

Cluster analysis allows for the examining of structural similarities or dis-similarities [15]. By combining the unsupervised nature of clustering with the supervised classification, one can correctly and reliably measure the relationship between the sensory systems and understand their behavior. Therefore, in situations where data can be classified, clustering can be performed between the groups to determine their structures (as a measure of their distinctness) and class labels assigned to evaluate the results of the clustering. Evaluating the clustering results using external measures such as purity, precision, recall, and F-measure can be valuable for understanding their characteristics [16]. Among various clustering methods, clustering using the Kohonen neural network (KNN, self-organizing map) produces an effective representation of the data, illustrating the hidden patterns inherent to the characteristics of the data, as a specific data distribution (e.g., normal) is not expected, non-linear relationships can be accommodated, the prior information about the number of clusters is not required and its learning algorithm provides a robust clustering performance [17,18]. The number of neurons and their topology in its output map (Kohonen or self-organizing map) can be set to best represent the data. 

The purpose of this study was to use the Kohonen neural network to investigate and demonstrate the effects of sensory systems to balance using their respective time-domain directional sway information. The subsequent sections are organized as follows: a brief introduction of the Kohonen neural network is provided, followed by the methodological section which is divided into data collection and analysis parts, and finally the results are explained.

## 2. Kohonen Neural Network

**Figure 1:**
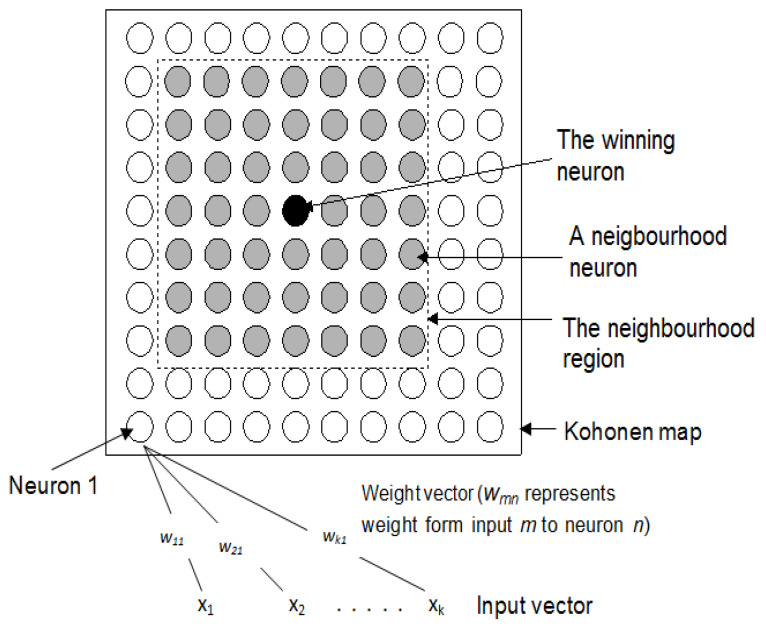
Kohonen neural network with a 10 × 10 output (Kohonen) map.

Developed by the Finnish researcher Tuevo Kohonen in the 1980s [19], the Kohonen neural network is a type of unsupervised learning that uses clustering to project an input space of high dimensions onto a low dimensional prototype regular grid that can be used to visualize and explore the relationship properties of the data [20]. The Kohonen network’s operation is based on competitive learning, i.e., a process whereby each neuron competes with other neurons to represent an input example (input vector) [21]. The Kohonen network has been widely used in data exploration applications [22]. Its use in balance and gait analysis has been reported in a number of studies. It was used to extract and visualize information about high dimensional balance strategies (full-body kinematics) before and after a 6-week slackline training intervention of thirteen young adult subjects (11 females and 2 males) [23]. The results showed that the balance coordination pattern between pre- and post-tests for the slackline task was significantly different [23]. Gait investigation of the data from 60 healthy normal subjects (mean age 63.3 years, age range 37 to 86 years, 47% men) and 60 patients (mean age 68.8 years, age range 45 to 84 years, 62% of men) with idiopathic Parkinson disease using Kohonen network showed that the identified groupings were consistent with the classification carried out by experts in function of traditional gait dynamic analysis [24]. Kohonen network has also been applied in areas of balance to identify movement patterns and variability among young and older adults [25]. Figure 1 shows a two-dimensional Kohonen map of size 10 × 10 neurons capable of projecting the characteristics of the input dataset.

The operation of the network as described in [21,22,23,24,25,26,27] is summarized using the following 4 steps:

i. Initialization

The learning process starts with initializing the weights associated with each neuron. A weight *w_mn_* is associated with the connection from *m^th^* feature of the input vector (representing an input example) to *n^th^* neuron. The number of weights for each neuron is the same as the number of features in the input vector. The weights can be initialized based on prior information or small randomly selected values close to zero. The learning rate and neighborhood size are also initialized (these are defined in step iii). Kohonen network requires the learning termination point to be initialized. The manners this can be achieved are explained in step (iii).

ii. Competition

Kohonen network is presented with the *j^th^* training input vector (*Xj*). Each neuron determines the closeness (or distance) of the current input vector to the weights associated with its connections. The neuron with the closest connection weights to the input vector (i.e., smallest distance) is selected as the winner. When the Euclidean distance is used to determine closeness, the distance (*d_n_*) for neuron *n* is

(1) $$d_{n}=\sqrt{{\left(x_{1}-w_{1n}\right)}^{2}+{\left(x_{2}-w_{2n}\right)}^{2}+\ldots +{\left(x_{k}-w_{kn}\right)}^{2}}$$

iii. Adaptation

This step can be divided into two tasks. Firstly, the neighbors of the winning neuron are identified and secondly, the weights of the winning neuron and those of its identified neighbors are updated. The neighboring neurons are located in the neighborhood region (shown in Figure 1) that surrounds the winning neuron. The size of the neighborhood region is defined as the number of neurons in each direction of the winning neuron. In Figure 1, the neighborhood size is 3 neurons. 

Adaptation involves updating the weight vectors of the winning neuron and its neighbors. This is carried out using the Kohonen learning rule,

(2) $$W_{j_{updated}}=W_{j_{current}}+\mu \left(X_{j}-W_{j_{current}}\right)$$

where ***W****_-(j_current)_* and ***W****_-(j_updated)_* represent the current and updated weight vectors for the winning neuron respectively. The learning rate (0 < μ ≤ 1) controls the amount of weights changes taking place during each learning iteration. Larger values of μ lead to faster learning but they may reduce adaptation effectiveness. Its initial value is typically chosen heuristically.

In order to improve learning, the weights associated with the neighborhood neurons are also updated to a lesser extent than the winning neuron. Typically, a Gaussian function is used to influence the extent of this update, with relatively (as compared to the winning neuron) smaller weights changes as neurons become further from the winning neuron. The initial size of the neighborhood region and learning rate can be reduced as learning progresses. This is to move from a coarse adjustment of the weights to finer adjustments.

iv. Termination of iterations

Steps (ii) and (iii) are repeated until either the required number of iterations is completed, or the magnitude of weight changes reaches a predefined value.

The Kohonen network can be implemented using sequential or batch processing. The difference between the sequential and the batch processing is that in the former, the neurons’ weights are updated after each input vector is presented to the network while for the batch processing the weights are updated after an epoch, i.e., a single pass over the entire input data set [28]. The advantages of the batch processing method include: no dependence upon the order in which the input data are presented since the weights updates are not recursive [28] and issues regarding poor convergence are eliminated as the learning rate coefficient is absent in the batch Kohonen network algorithm [29].

## 3. Methodology

### 3.1. Accelerometry Algorithm to Analyse Postural Sway

**Figure 2:**
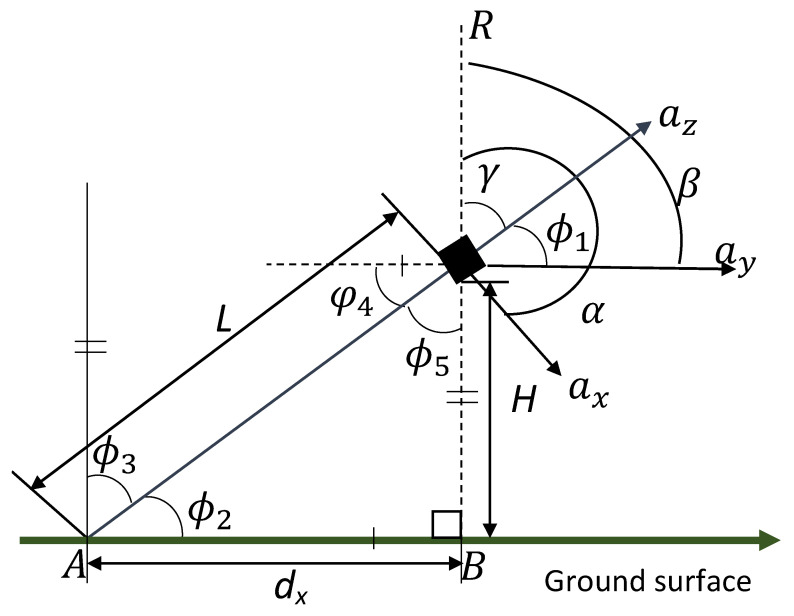
Sway projection based on the inverted pendulum [30].

The analysis of postural sway involved the projection of the body movements at the center of mass (COM) to the ground surface. This could be achieved by considering the principle of the inverted pendulum [30] as shown in Figure 2.

*L* represents the distance of the COM position to the ground which is assumed to be constant for each individual. The resultant acceleration is shown as *R*. The angles made by each directional acceleration *a_x_*, *a_y,_* and *a_z_* are shown as α, β, and γ respectively. They are obtained from their directional cosines cos(α), cos(β), and cos(γ) respectively. The angles φ_1_, φ_2_, φ_3_, φ_4_, and φ_5_ are used for mathematical justifications. The ground displacements from the origin in the *x* and *y* directions are *d_x_* and *d_y_* respectively and *H* represents the ground projection of the COM height. Equations (3)–(5) describe the algorithm’s operation. The units for distance, acceleration and angle were chosen as centimeters (cm), centimeters per second square (cm/s^2^), and degrees (°) respectively.

(3) $$R=\sqrt{a_{x}{}^{2}+a_{y}{}^{2}+a_{z}{}^{2}}$$

(4) $$\cos\left(\alpha \right)=\frac{a_{x}}{R},\;\;\;\cos\beta =\frac{a_{y}}{R},\;\;\cos\left(\gamma \right)=\frac{a_{z}}{R}$$

(5) $$d_{x}=-L\cos\left(\alpha \right),\;\;d_{y}=-L\cos(\beta ),\;\;H=L\cos(\gamma )$$

### 3.2. Accelerometry Device Used for Data Recording

**Figure 3:**
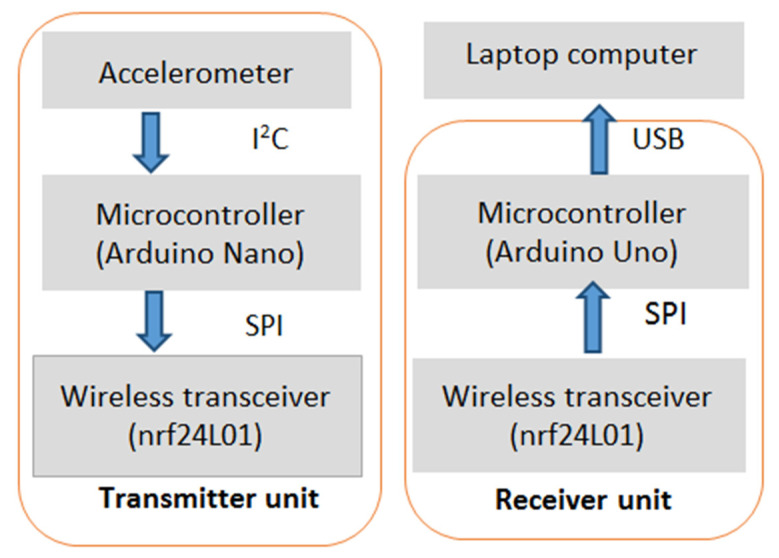
Data recording transmitter and receiver units.

Figure 3 shows the device developed for the accelerometry data recordings. It consisted of transmitter and receiver units.

The transmitter unit consisted of a microcontroller (type: Arduino Nano), an inertia measurement unit (IMU, type: MPU 6050), and a wireless transceiver (type: nRF24L01). It was responsible for measuring the subject’s postural sway. Communication between the IMU and the microcontroller was facilitated using the inter-integrated circuit (I2C) protocol. The IMU signals utilized and processed in the study were the IMU’s accelerometer signals, represented by *x*, *y*, and *z*. Each signal indicated the movement in one of the 3 orthogonal directions. Communication between the wireless transceiver and the microcontroller was via the serial peripheral interface (SPI) protocol. The signals were wirelessly sent to the receiver unit by the transceiver.

The receiver unit was responsible for receiving the signal from the transmitter unit and transferring the information to a laptop computer for storage. It consisted of a microcontroller (type: Arduino Uno) and a wireless transceiver (type: nRF24L01). It was interfaced with the laptop computer using a USB cable.

### 3.3. Participants’ Details and Experimental Procedure

**Figure 4:**
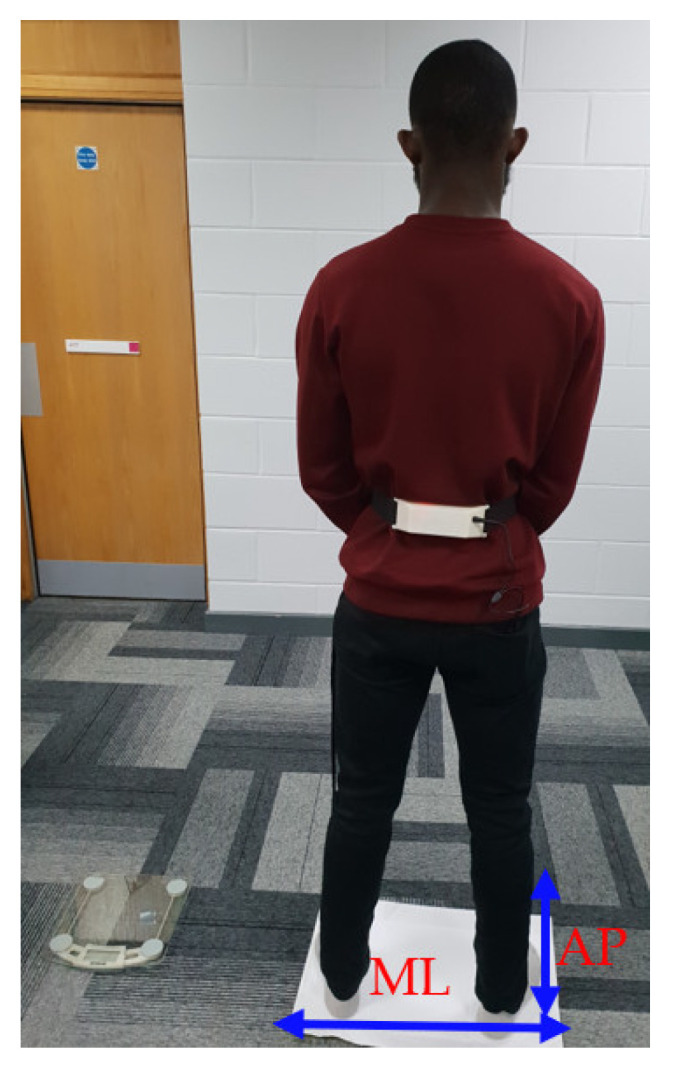
A subject performing condition 3 of the mCTSIB with a white sponge under his feet and the accelerometer transmitter unit (the white box) worn at the lower back.

Twenty-three healthy adult subjects (10 males and 13 females), mean (standard deviation) age: 24.5 (4.0) years, mean height (standard deviation): 173.6 (6.8) cm, mean weight (standard deviation): 72.7 (9.9) kg, with no previous history of balance dysfunction, participated in the study. During recording, the subject stood relaxed while looking at a wall at a distance of 1 m. The transmitter unit was strapped to the subject’s lower back, at approximately the iliac crest, as shown in Figure 4. The data acquisition software was written in the Processing language that is compatible with Arduino microcontroller boards. The data recording lasted for two minutes with 30 s allocated for each of the four conditions of the mCTSIB. A longer duration recording was avoided to ensure the subjects did not become tired. The four conditions of mCTSIB involved the subject: (i) standing on a firm surface (ground) with eyes open, (ii) standing on a firm surface with eyes closed, (iii) standing on a flexible surface (a sponge with dimensions: 10 cm height, 0.5 m length and width), with eyes open, and (iv) standing on the flexible surface with eyes closed. The signal sampling rate was 60 samples per second. The subjects declared not to have ingested any substance capable of affecting their balance 48 hours prior to the recordings and ethical permission was obtained from the university ethical committee prior to conducting the recordings.

### 3.4. Data Analysis

The analysis had three stages: (i) processing the accelerometry raw data into sway information, (ii) clustering the sway information, and (iii) interpreting the clustering results to determine the information provided by ML and AP sway directions.

#### 3.4.1. Conversion of the Raw Accelerometry Data into Sway Information

The raw accelerometry data used for carrying out the analysis consisted of the body’s acceleration in the three axes (*x*, *y* and *z*) of the tri-axial accelerometer. The raw accelerometer data were converted into accelerations in units of gravity (g) by dividing the digital output into units of least significant bits (LSB) of the tri-axial accelerometer by the accelerometer’s sensitivity scale factor in units of LSB/g. The accelerometer’s sensitivity scale factor for a full-scale range of ±2 g was 16,384 (LSB/g). Furthermore, the acceleration outputs (i.e., signals representing acceleration in *x*, *y*, and *z* directions) were lowpass filtered using a second order lowpass Butterworth filter with a cut of frequency of 4 Hz. The resultant acceleration (*R*), the directional cosines: cos(α) cos(β) and cos(γ) and the displacement in the *x* and *y* axes from the origin (position), *d_x_* and *d_y_* were obtained using Equations (3)–(5). The subject’s body position, velocity, and acceleration were obtained using Equations (6) and (7), where $D_{k}{}_{n}$, $V_{k}{}_{n}$, and $A_{k}{}_{n}$ are the positional displacements, velocity, and acceleration respectively, $D_{k}{}_{RMS}$, $V_{k}{}_{RMS}$, and $A_{k}{}_{RMS}$ are their corresponding root mean square (RMS) values, *k* corresponds to the direction of interest, i.e., ML and AP, $d_{k}{}_{1}$ is the first term used to remove the inclination offset on the subject’s back.

Care was taken to ensure the transmitter unit was attached to each subject in a consistent manner. The RMS measurements were utilized due to their effectiveness in differentiating between the four conditions of the mCTSIB, established in an earlier study [30].

(6) $$D_{k}{}_{n}=d_{k}{}_{n}-d_{k}{}_{1},\;\;\;\;V_{k}{}_{n}=\frac{D_{k}{}_{n}-D_{k}{}_{n-1}}{T},\;\;\;\;A_{k}{}_{n}=\frac{V_{k}{}_{n}-V_{k}{}_{n-1}}{T}$$

(7) $$D_{k}{}_{RMS}=\sqrt{\frac{1}{N}\overset{N}{\underset{n=1}{\sum }}{\left(D_{k}{}_{n}\right)}^{2}},\;V_{k}{}_{RMS}=\sqrt{\frac{1}{N}\overset{N}{\underset{n=1}{\sum }}{\left(V_{k}{}_{n}\right)}^{2}},\;\;\;A_{k}{}_{RMS}=\sqrt{\frac{1}{N}\overset{N}{\underset{n=1}{\sum }}{\left(A_{k}{}_{n}\right)}^{2}}$$

#### 3.4.2. Clustering of the Postural Sway Information

Correlation analysis was carried out among the variables of the ML and AP directions, i.e., the RMS measures of position, velocity, and acceleration, to identify the variables that had strong correlations with each other. Highly correlated variables were defined by the coefficient of correlation being equal to, or greater than, 0.85 [31]. Prior to carrying out correlation analysis, a test was carried out to establish whether the data were from a normal distribution with the level of significance (α) set to 5%. The choice of correlation to apply was dependent on the result of the test of normality of the variables [32]. In situations where the measure deviated from normality, Kendall’s tau correlation coefficient was used to compute the correlation, otherwise, Pearson correlation was used.

The clustering of the input data was carried out in two abstraction levels. The two-level approach has been shown to be computationally more efficient than using only the K-means clustering [20] as only the representatives of the prototype vectors, i.e., the centroids, are used for the clustering. The first abstraction level involved using the Kohonen network to cluster the uncorrelated variables thereby forming a set of prototype vectors. The Matlab^©^ (MathWorks^®^, Natick, MA, USA) batch algorithm of the Kohonen network was used for training with a Kohonen map size of 10 by 10 neurons (total 100 neurons). A large map was used to explore the relationships between the four conditions of mCTSIB. The default value of the initial neighborhood size (i.e., 3 neurons) was used for the training and the number of training iterations was set to 1000. The entire dataset was used as the training and test set because the interest was to explore the interactions between the conditions of the mCTSIB. Each mCTSIB condition consisted of the data from the 23 subjects and the clustering of the data set was conducted by pairing each condition with condition 1, taking condition 1 as the reference (as condition 1 incorporated all the balance-related sensory systems). Therefore, three examination combinations could be carried out, i.e., conditions: 1 and 2, 1 and 3, and 1 and 4. 

The second abstraction level consisted of the clustering of the formed Kohonen network prototype vectors. For this purpose, the clustering was performed using the K-means algorithm. The K-means algorithm divides the data set into K clusters such that the within cluster’s sum of the square is minimized [33]. The built-in K-means algorithm in the Matlab (MathWorks^®^, Massachusetts, USA) batch was used for the implementation of the K-means clustering with the default distance metric, i.e. Euclidean. The processes involved were representing the porotype vectors formed during the first abstraction level by their centroids and then clustering the resulting centroids. The prototype vectors represented the neurons associated with at least one input data. The main issue that needed considering when performing the K-means algorithm, was determining the number of clusters. To this effect, the number of clusters was determined by performing K-means clustering on the resulting centroids of the prototypes formed during the first abstraction level for the different number of clusters (K) varying from 2 to 30 and evaluating the resulting cluster separation, using the Davies-Bouldin (DB) index as the measure of the clustering separation. The DB index is an internal evaluation measure based on the ratio of within to between cluster separations [34]. Usually, the lower the value of the DB index, the better the clustering performance. The DB index was determined using Equation (8).

(8) $$DB=\frac{1}{k}\overset{k}{\underset{i=1}{\sum }}DB_{i},\;\;\;\;\;\;\;\;DB_{i}=maxDB_{ij},\;\;\;\;\;\;\;\;DB_{ij}=\frac{\sigma_{c_{i}}+\sigma_{c_{j}}}{\left||\mu_{c_{i}}-\mu_{c_{j}}|\right|}$$

where $\sigma_{c_{i}}$ is the standard deviation of cluster *c_i_*, $\sigma_{c_{j}}$ is the standard deviation of cluster *c_j_*, $\mu_{c_{i}}$ is mean of cluster *c_i_*, $\mu_{c_{j}}$ is mean of cluster *c_j_*, *DB_ij_* is an array of DB indices for cluster *i* with respect to the *j^th^* cluster, where i ≠ j, j = i + 1:k, *DB_i_* is DB index for the *i^th^* cluster. The number of clusters with the minimum DB was used for external evaluation. 

The minimum DB may not be optimal [20], thus further analysis of the clustering was performed at other local minima, and the number of clusters (K) for the *K-means* clustering, selected based on the highest F-measure. The DB index selected from the clustering between conditions 1 and 4 was used for the clustering between conditions 1 and 2, and conditions 1 and 3 as the greatest differences were expected to be between conditions 1 and 4.

#### 3.4.3. Clustering Performance

After clustering the prototype centroids into K clusters, the centroids were replaced by their actual constituent data points that were obtained from the first abstraction level. The class labels corresponding to the four conditions of the mCTSIB were assigned to the data points of the formed clusters. For evaluation purposes, each cluster was labeled by most of the categories present in the cluster and also a labeled category was assigned to more than one cluster if there was a dominant grouping in that cluster. External evaluation measures such as purity, precision, recall, and F-measure were utilized to measure the differences between the four conditions of the mCTSIB. The purity measure evaluated the coherence of a cluster, i.e., the degree to which a cluster contained data entries from a single category [35]. The greater the purity measure, the more the separation between the categories. The precision of a cluster is the same as its purity. The recall of a cluster measures the fraction of points of the majority partition shared in common with the cluster. Since the analysis had been limited to non-empty clusters (i.e., clusters with at least one entry), the lowest amount of purity would be obtained, when all the samples converged into a single cluster. Provided the numbers of samples between the classes were equal, the samples of one class divided by the total number of samples of all the classes gave the lowest purity. In this case, the lowest purity that could be obtained was 0.5 since there were 23 samples in each group and the clustering consisted of two groups (46 samples), i.e., 23 divided by 46. Similarly, the lowest clustering precision was obtained when all the data samples were present in one cluster. In this case, since there were 23 samples in each group and the clustering consisted of two groups (46 samples), the minimum precision was 0.5, i.e., 23 divided by 46. The minimum recall would exist when only one sample would be present in each cluster. That is, the recall of a clustering varies inversely with the number of clusters, i.e., 0.5 (1 divided by 2) for two clusters, 0.25 (1 divided by 4) for four clusters, 0.17 (1 divided by 6) for six clusters, etc. The formulae of these measures are presented below:

(9) $$purity=\overset{r}{\underset{i=1}{\sum }}\frac{n_{i}}{n}purity_{i},\;\;\;\;purity_{i}=\frac{1}{n_{i}}\begin{array}{c} k\\ \max\\ j=1 \end{array}\left\{n_{ij}\right\}\;$$

(10) $$prec_{i}=\frac{1}{n_{i}}\begin{array}{c} k\\ \max\\ j=1 \end{array}\left\{n_{ij}\right\}=\frac{n_{ij_{i}}}{n_{i}}$$

(11) $$recall_{i}=\frac{n_{ij_{i}}}{m_{j_{i}}}$$

(12) $$F_{i}=\frac{2\times prec_{i}\times recall_{i}}{prec_{i}+recall_{i}}=\frac{2n_{ij_{i}}}{n_{i}+m_{j_{i}}}\;$$

where *n_ij_* is the number of elements of class *j* in the *i^th^* cluster, *n_i_* is the total number of elements in the *i^th^* cluster, *n* is the total number of elements of the dataset, *purity_i_* is the *i^th^* purity of the clustering. $n_{ij_{i}}$ is the maximum elements of the classes in the *i^th^* cluster, *prec_i_* is the *i^th^* precision. $m_{j_{i}}$ is the number of elements of the resulting maximum *j^th^* class of the *i^th^* cluster, *recall_i_* is the recall of the *i^th^* cluster. The F-measure of the clustering was obtained by taking the average over all the clusters as

(13) $$F=\frac{1}{r}\overset{r}{\underset{i=1}{\sum }}F_{i}\;\;$$

*F_i_* is the F-measure of the *i^th^* cluster. More details about these measures are available [36].

The clustering process was repeated thirty times, with the data randomized at each repetition and its corresponding external measures recorded and utilized for further analysis. The repetition of the clustering 30 times was conducted to guarantee the reliability of the values. A statistical test was carried out on the recorded external measures between the ML and AP directions to determine whether a significant difference existed between their results. The choice of statistical test for the significant difference was subject to the outcome of the test of normality from the external measures. In cases the outcome indicated data were from a normal distribution, the independent sample t-test was used otherwise the Mann-Whitney U-test was used. The software program for the external evaluation of the clustering performance was writing in Matlab^©^ (version 2017a, MathWorks^®^, Massachusetts, USA) batch while the statistical test was carried out using SPSS^®^ (version 24, IBM, Armonk, NY, USA).

## 4. Results

### 4.1. Correlation Result

**Figure 5:**
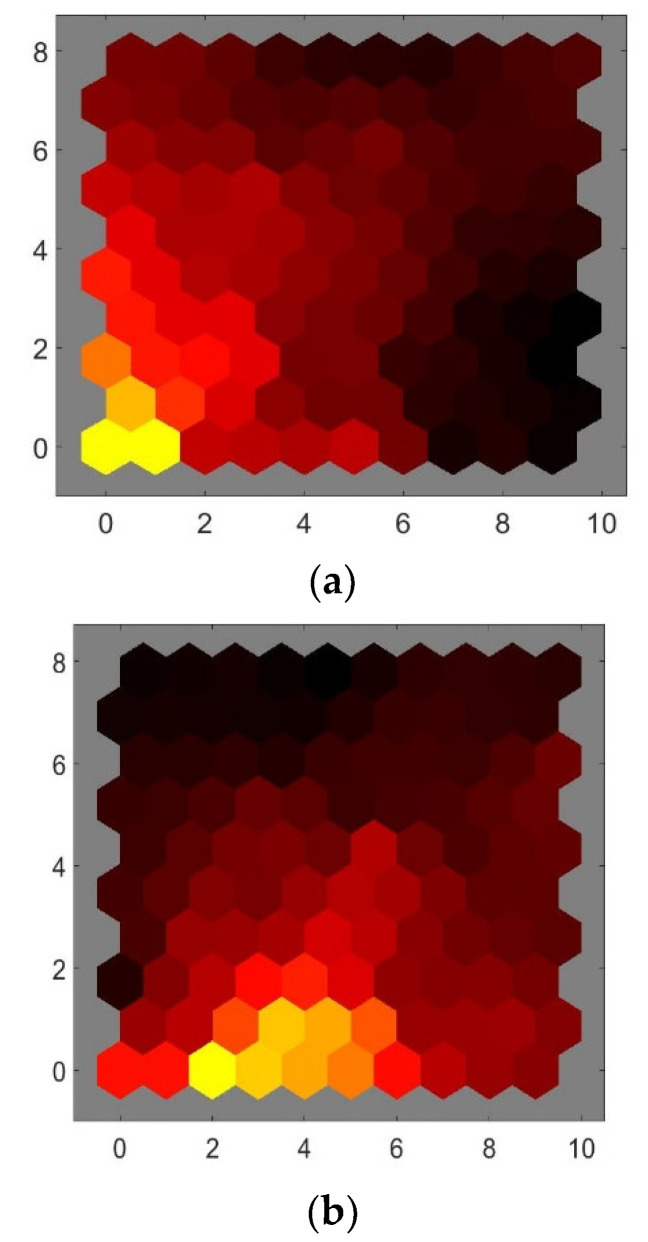

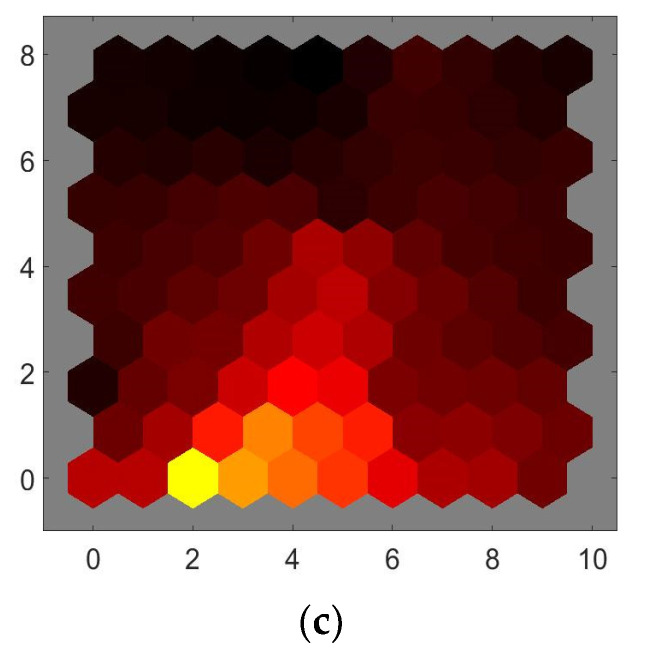
Correlation between the variables using the Kohonen network weight planes. (**a**) RMS position, (**b**) RMS velocity, and (**c**) RMS acceleration respectively. The horizontal and vertical axes are neurons’ positions.

The correlations between the variables, i.e., the RMS values of position, velocity, and acceleration as examined using the weights of the Kohonen network’s neurons (weights planes) are shown in Figure 5. The black color represents the most negative connections, red represents no connection, and yellow represents the strongest positive connections. The weights planes are used as an indication of correlation between the input variables, where closely related patterns represent strong correlations. Similar colors of weights can be observed between the RMS velocity and the RMS acceleration, indicating a strong correlation between the two variables. The result of the Shapiro-Wilk test of normality indicated that the variables were not from a normal distribution (*p* < 0.05). Thus, Kendall’s tau correlation was conducted between the variables and the result suggested that there was a strong positive correlation between the RMS velocity and RMS acceleration (r_τ_ (21) = 0.929, *p* < 0.01) while a significant weak positive correlation existed between the RMS position and RMS velocity r_τ_ (21) = 0.344, *p* = 0.022), and the RMS position and RMS acceleration (r_τ_ (21) = 0.32, *p* = 0.032) with the level of significance (α) equal to 5%. Thus, RMS position and velocity were used for the subsequent analysis.

### 4.2. Clustering Results

**Figure 6:**
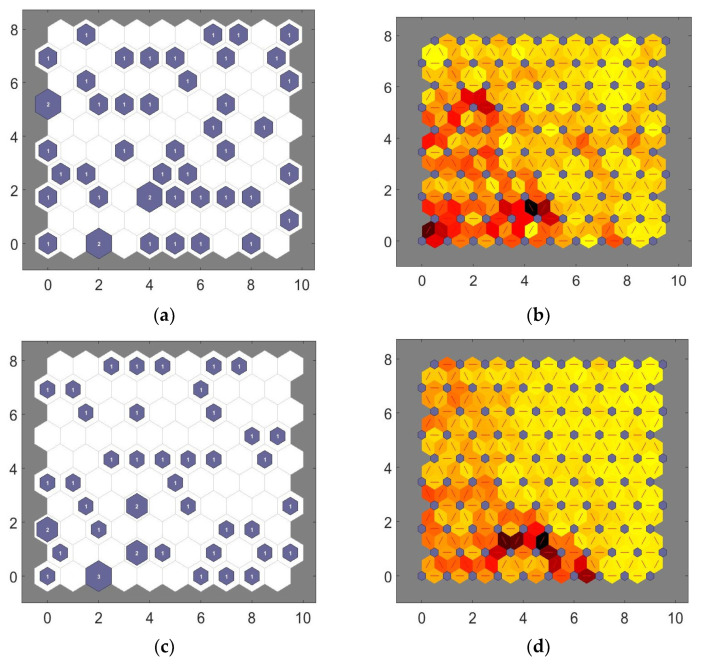
Plot of neighborhood distance and input vector hits of conditions 1 and 4. (**a**,**b**) representing the AP direction, (**c**,**d**) representing the ML direction. The horizontal and vertical axes are neurons positions.

The visualization of the Kohonen map and its structure using the test set for conditions 1 and 4 for both the AP and ML directions is shown in Figure 6.

Figure 6a shows a larger section of darker colors in the neighborhood distance plot of the AP direction as compared to that obtained from the ML direction as shown by Figure 6c. These darker colors indicate that the clustering obtained by using the measures of the RMS position and velocity of the AP direction appears to be coarser as compared to those obtained from the corresponding measures of the ML direction. However, the spread of the brighter colors throughout the map as shown by the neighborhood distance indicates a poor clustering between the sensory systems as expected for healthy adult subjects. Nevertheless, the greater coarseness of the map in the AP direction suggests larger sway differences between the sensory systems as compared to the sway differences indicated by the ML direction. This shows the direction’s ability to differentiate between the sensory system(s) associated with sway, and in turny, indicates the sensitivity of the direction, i.e., the variation of the sensory system(s), results in the largest sway toward the AP direction.

**Figure 7:**
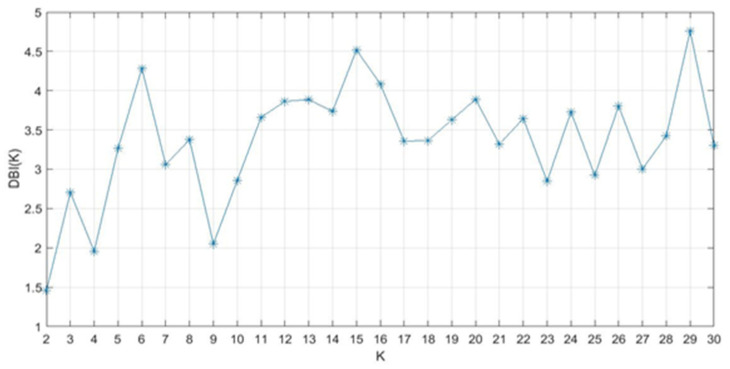
Davies-Bouldin index values for clusters K, showing K = 2 as the minimum.

The results of the DB index for clusters K = 2 to 30 are plotted in Figure 7. The figure indicates the average of 30 repetitions for conditions 1 and 4 with K = 2 to be lowest, other local minima exist at K = 4 and 9.

The mean F-measures obtained for the AP direction between conditions 1 and 4 for 30 repetitions for the number of clusters (K) equal to 2, 4, and 9 were 0.717, 0.448, and 0.249 respectively. Similarly, the mean F-measure obtained for the ML direction between conditions 1 and 4 for K = 2, 4, and 9 clusters were 0.532, 0.356, and 0.246 respectively. Since the values of the F-measure for two clusters were higher than those of four and 9 clusters, two clusters were used for further analysis.

**Figure 8:**
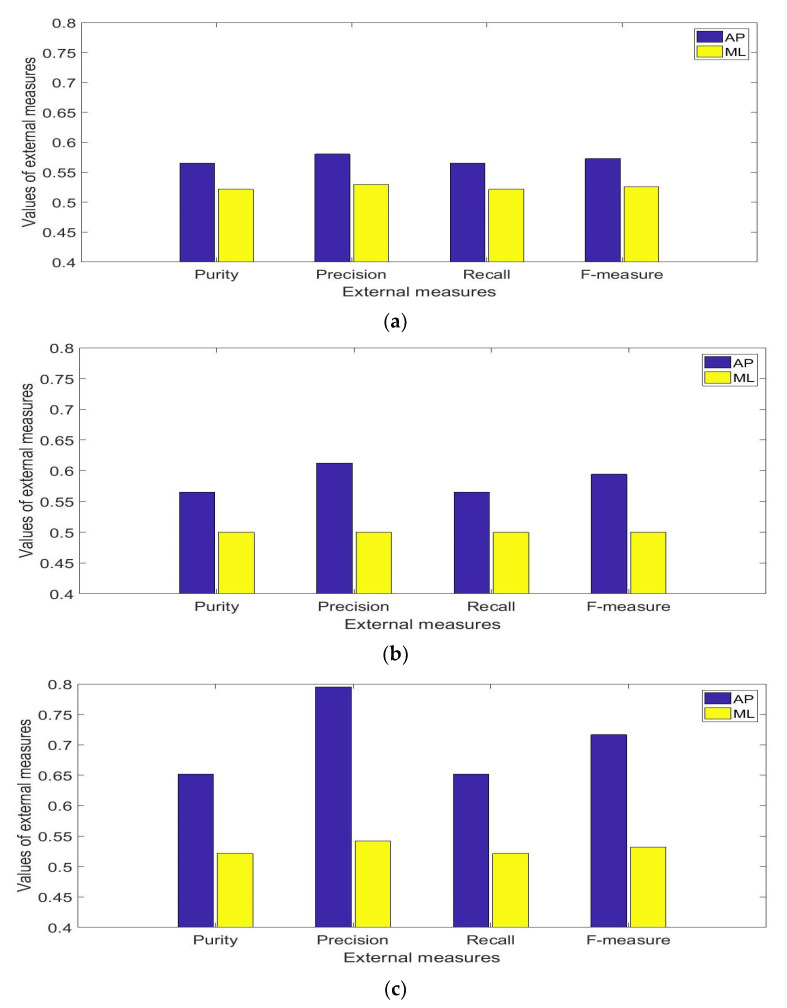
Results of median values of the external measures over 30 repetitions: (**a**) mCTSIB conditions 1 and 2, (**b**) mCTSIB conditions 1 and 3 (**c**) mCTSIB conditions 1 and 4.

Using two clusters for the K means clustering, the averages of the respective clustering performance measures between conditions 2, 3, and 4 of the mCTSIB and condition 1 are shown in Figure 8. The blue bars represent the AP direction, and the yellow bars represent the ML direction. The precision and F-measure for the AP direction appeared lower for Figure 8a (conditions 1 and 2) than it is for Figure 8b,c. This indicates that the presence of the proprioceptive system reduces AP direction of sway. Conversely, all the external measures for the ML direction were lowest for Figure 8b as compared to Figure 8a,c respectively. The median values and interquartile ranges of these measures for 30 clustering repetitions are shown in Table 1.

Similar values of the external measures would have been observed if the sway in the ML and AP directions were to be the same. On average, over all the four mCTSIB conditions, the purity, precision, recall, and F-measure for the AP direction were respectively 7.12%, 11.64%, 7.12%, and 9.5% higher than the respective values obtained from the ML direction. The Mann-Whitney U-test showed that significant differences (*p* < 0.05) existed between the corresponding results of the external measures obtained from each direction. The results of these measures are representative of healthy adult subjects and thus the results may differ across subjects with balance problems.

**Figure 9:**
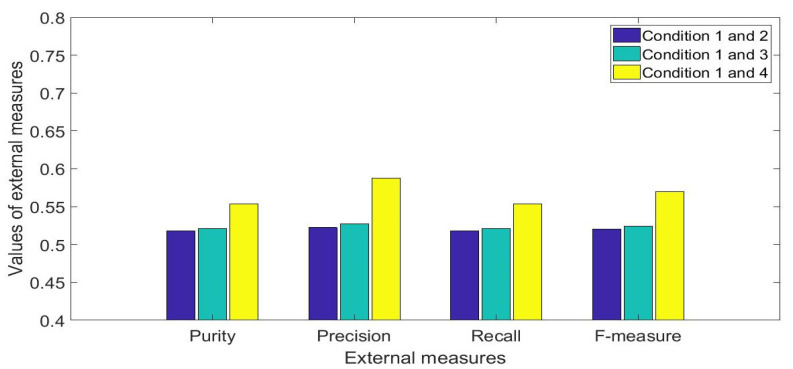
Results of the median values of external measures for the combination of the ML and AP directions for 30 repetitions.

When the RMS variables of the ML and AP sway directions were combined, the order of similarity was the same as that obtained using the AP direction for similarity measurement. The clustering results are shown in Figure 9.

## 5. Discussions

This study was designed to investigate, for diagnostic purposes, the effect of the balance-related sensory systems to balance for young healthy adult subjects in a well-lit environment, (10 males and 13 females) with a mean (standard deviation): ages 24.5 (4.0) years, height: 173.6 (6.8) centimeter, and weight: 72.7 (9.9) kg, by examining the clustering characteristics of their sway in the mediolateral (ML) and anterior-posterior (AP) directions.

Poor clustering was observed using the sway from the ML and AP directions across the four conditions of the mCTSIB as the sway was from a cohort of healthy adult subjects. However, disparities existed in clustering results from the respective directions with a reduced similarity in the AP direction as compared to the ML direction. This was an indication of sway differences and highlights that as the sensory systems were reduced/altered, i.e., from condition 1 to 4 of the mCTSIB, an increase in the sway occurred with more sway in the AP direction than the ML direction. Therefore, the AP direction was more sensitive to the responses of the sensory systems. Dependencies on the sensory systems to balance for the subjects using the AP direction indicated that the subjects depended more on the proprioceptive system than the visual system. Contrarily, considering the ML direction, the reverse was the case. A combination of the ML and AP sway reduced the differences between sensory systems, although the result still suggested that more reliance was placed on the proprioceptive system than the visual system. A similar result was reported in [1,37], that in a well-lit environment with a solid base of support, healthy individuals depended more on the somatosensory system for balance. A combination of the sway from both directions suggested that the results of the differences are affected as the direction with less sway tends to cancel the direction with more sway or vice-versus. Consequently, for accurate analysis of balance, the study indicated the directional sensitivity of sway should be considered with more emphasis placed on the direction that represents more sway. A larger sway does not necessarily imply the direction with the largest magnitude of sway but the direction that represents the COM sway for a longer time interval.

The presence or absence of the proprioceptive system as indicated by the comparison between the clustering results of conditions 1 and 2 and conditions 1 and 4, affected the AP directional sway but had only a minor effect on the ML sway as indicated by their clustering results with a value of 0.5 being the lowest attainable value for the external measures. This indicated that the proprioceptive system had only a minor effect on the ML sway. In contrast, the presence or absence of the visual system as indicated by the comparison between the clustering results of conditions 1 and 3 and conditions 1 and 4, affected both the AP and ML directional sway as indicated by their clustering results with a value of 0.5 being the lowest attainable value for the external measures. This indicated that the visual system in addition to reducing the AP directional sway, had a significant effect on the ML directional sway as it completely removed any sway differences. A similar result was reported in [38] with a different cohort of subjects. In [38], it was reported that ML sway dramatically increased, when the subjects stood on a sponge surface with their eyes closed, than when they stood, with their eyes open thus indicating that older subjects relied on the visual system to correct ML postural sway. Thus, we suggest with addition to a reduction in AP sway, a reduction in ML sway should particularly be used to assess the functionality of the visual system while a reduction in AP sway should be used in particular for the proprioceptive and vestibular system.

In general, the clustering results revealed that in healthy adult subjects, the sensory systems sway is highly related. Thus, emphasis should be placed on training programs that ensure the full operation of all the systems. In the case where full operation cannot be guaranteed, the interaction of the visual or the proprioceptive with the vestibular can help to achieve functional balance. The importance of the vestibular system has been reported in several studies. In [39], patients with bilateral complete loss of peripheral vestibular system function, were reported not to be able to engage in activities that required an intact vestibular system such as sports and household activities. Similarly, a home-based exercise program was found in [40] to significantly improve balance abilities in individuals with chronic vestibular dysfunctions. Since with the vestibular system only, no large difference was observed nor was there any visible fall, we suggest that intervention to ensure the proper functioning of the vestibular system should be of paramount importance.

The study’s findings can be summarized as:

i. The clustering of the postural sway, based on the RMS measures of the body’s position and velocity of the AP direction, showed larger values of external measures of the clustering performances as compared to similar variables from the ML direction. As a result, it may be inferred that the AP direction was more sensitive to the effect of the information of the sensory system as compared to the ML direction.
ii. Hindrance in the operation of the visual system leads to an increase in the external performance measures of the clustering in the ML direction. In clustering between the eyes open conditions (conditions 1 and 3), the clustering evaluation measures, i.e. purity, precision, recall, and F-measure were 0.5, which was equal to the minimum value that could occur from the clustering. Thus, postural sway in the ML direction is a characteristic of the contribution of the visual system.
iii. Using separate directions resulted in differing order of similarities across the four conditions of the mCTSIB. When the clustering measures of the AP direction were used for analysis, the order of similarities of the conditions were conditions 1, 2, 3, and 4. However, when the clustering measures of the ML direction were used, the order of similarities were conditions 1, 3, 2, and 4 and the results showed less disparity across the conditions. When the measures from the AP and ML directions were combined, the external clustering performance measures were reduced. This indicated that combining the results of ML and AP directions results in closer similarities between the conditions.
iv. There was not a large variation between the maximum and the minimum (0.5) values across all external measures of the clustering performance between the conditions of the mCTSIB using the RMS values of the position and velocity of the respective ML and AP directions. The maximum value of the external measures corresponded to the value of the precision measure (0.795) for the clustering between the conditions 1 and 4. In the case of clustering with two clusters, the minimum and maximum values of the external measures that can occur for two groups with an equal number of samples are 0.5 and 1 respectively. Thus, the difference of 0.295 between the precision value and the minimum i.e. 0.795 minus 0.5 is considered small. Therefore, it was concluded that for healthy young adult subjects, there is a strong interrelationship between the mCTSIB conditions and their postural sway results cannot be clustered well into two distinct groups.

## 6. Conclusions

For diagnostic purposes, the effect of the sensory systems in balance obtained from the mediolateral (ML) and anterior-posterior (AP) directions of 23 healthy young adults (10 males and 13 females) with closely related age, height, and weight were investigated. The subjects engaged in the four conditions associated with the modified Clinical Test of Sensory Interaction and Balance (mCTSIB). The measurements of the root mean square sway values of body position and velocity obtained from an inertial measurement unit (IMU) placed at the lower back of the subjects at approximately the position of the iliac crest.

Their effects were investigated using Kohonen neural network clustering performances which were evaluated using external measures such as purity, precision, recall, and F-measure. The results indicated that the information provided by ML and AP directions in balance analysis differ and that the direction of sway plays an important role in correctly assessing a subject’s balance. In particular, it was observed that the AP direction performed better than the ML direction in clustering the data from the four conditions associated with the mCTSIB. The ML sway was characterized by the hindrance of the visual system. The combination of the sway variables from the ML and AP directions resulted in smaller differences between the mCTSIB conditions. We suggest with addition to a reduction in AP sway, a reduction in ML sway should particularly be used to assess the functionality of the visual system while a reduction in AP sway should be used in particular for the proprioceptive and vestibular systems.

## Figures and Tables

**Table 1:** Results of the median (interquartile range) values of the external measures for 30 clustering repetitions.

mCTSIB Conditions	Purity		Precision		Recall		F-Measure	
	ML	AP	ML	AP	ML	AP	ML	AP
1 and 2	0.522 (0.022)	0.565 (0.065)	0.530 (0.035)	0.58 (0.063)	0.522 (0.022)	0.565 (0.065)	0.526 (0.028)	0.573 (0.059)
1 and 3	0.500 (0.022)	0.565 (0.065)	0.500 (0.030)	0.613 (0.070)	0.500 (0.022)	0.565 (0.065)	0.500 (0.026)	0.594 (0.061)
1 and 4	0.522 (0.022)	0.652 (0.044)	0.542 (0.055)	0.795 (0.014)	0.522 (0.022)	0.652 (0.044)	0.532 (0.040)	0.717 (0.033)
Averages	0.515 (0.022)	0.594 (0.058)	0.524 (0.040)	0.662 (0.049)	0.515 (0.022)	0.594 (0.058)	0.519 (0.031)	0.628 (0.051)

## Data Availability

The subjects’ data recordings have restricted access.
